# Supplementary material for: A systematic review on the influence of coagulopathy and immune activation on New Onset Atrial Fibrillation in patients with sepsis
Source: PLoS One. 2025 Jan 29;20(1):e0318365. doi: 10.1371/journal.pone.0318365 (PMC11778662; doi:10.1371/journal.pone.0318365)
Supplement: S1 Table — (DOCX) [file pone.0318365.s001.docx]

S1 Table – Complete Search Strategy by Database

| Database | Search Strategy |
| --- | --- |
| Medline | 1. Sepsis/ (map term to subject heading)  2. (Sepsis, septic, septicemia, Toxemia, Toxaemia, Bacteraemia, Bacteremia) ab, ti.  3. 1 OR 2  4. Inflammation/ (map term to subject heading)  5. (Inflammation, inflamm*, CRP, “C-reactive protein“, ESR, "Erythrocyte sedimentation rate“, "plasma viscosity“, "procalcitonin”, PCT, IL-6, IL-1, "Tumour Necrosis Factor alpha“, "Serum amyloid A“, SAA, "heat shock proteins“, HSPs, TNF, "Tumour Necrosis Factor“, IL-10, "acute phase proteins“, ferritin, "soluble IL-2 receptor“, "white cell count“, monocyte, basophil, lymphocyte, eosinophil, neutrophil, "neutrophil-lymphocyte ratio“, "neutrophil lymphocyte ratio“, "systemic inflammatory response index") ab, ti.  6. 4 OR 5  7. Disseminated Intravascular Coagulation/ (map term to subject heading)  8. Blood Coagulation/ (map term to subject heading)  9. (Coagulopathy, "sepsis induced coagulopath*“, "SIC“, "disseminated intravascular coagulation“, "DIC“, "sepsis associated coagulopath*“, "SAC“, "coagulation marker*“, fibrinogen, prothrombin, "antithrombin III“, beta-Thromboglobulin, "beta Thromboglobulin“, "von Willebrand Factor*“, Thrombin, "Blood Coagulation Factor*“, "Factor V“, calcium, "factor IV“, "Factor VII“, "Factor VIII“, "Factor IX“, "Factor X“, "Factor XI“, "Factor XII“, "Factor XIII“, fibrin, "antifibrinolytic agent*“, "platelet count“, "Mean Platelet Volume“, P-Selectin, “P Selectin“, thromboplastin, "protein C“, "protein C inhibitor*“, "protein S“, thrombomodulin, "plasminogen inactivator*“, "plasminogen activator*“, plasminogen, carboxypeptidases, "thrombin-activatable fibrinolysis inhibitor“, TAFI, "fibrinopeptide A“, "fibrinopeptide B“, hemostat*, haemostat*, thrombocyt*, platelet*, D-dimer*, "tissue factor*“, thrombophilia, thrombocytopenia, PT, "prothrombin time“, aPTT, "activated partial thromboplastin time“, INR, "international normalised ratio“, "APTT Ratio“, "vitamin K“, "fibrin degradation products”, FDP, "fibrin split products" OR FSP, "Plasminogen activator inhibitor-1“, PAI-1, "Alpha-2 plasmin inhibitor“, "antiplasmin“, thromboelasto*) ab, ti.  10. 7 OR 8 OR 9  11. (coagul* AND marker*) ab, ti.  12. 10 OR 11  13. Atrial Fibrillation/ (map term to subject heading)  14. ("atrial fibrillation”, "AF”, "new onset atrial fibrillation”, new-onset-atrial-fibrillation, "NOAF“, "atrial flutter*“, "supraventricular tachycardia“, supraventricular-tachycardia, "supraventricular arrhythmia“, supraventricular-arrhythmia, "SVT“, "atrial tachycardia") ab, ti.  15. 13 OR 14  16. 6 OR 12  17. 3 AND 15 AND 16 |
| Cochrane Library | 1. MeSH descriptor: [Sepsis] explode all trees  2. (sepsis OR septic OR septicemia OR Toxemia OR Toxaemia OR Bacteraemia OR Bacteremia):ti,ab,kw (Word variations have been searched)  3. 1 OR 2  4. MeSH descriptor: [Inflammation] explode all trees  5. (inflammation OR inflamm* OR CRP OR "C-reactive protein" OR ESR OR "Erythrocyte sedimentation rate" OR "plasma viscosity" OR "procalcitonin" OR PCT OR IL-6 OR IL-1 OR "Tumour Necrosis Factor alpha" OR "Serum amyloid A" OR SAA OR "heat shock proteins" OR HSPs OR TNF OR "Tumour Necrosis Factor" OR IL-10 OR "acute phase proteins" OR ferritin OR "soluble IL-2 receptor" OR "white cell count" OR monocyte OR basophil OR lymphocyte OR eosinophil OR neutrophil OR "neutrophil-lymphocyte ratio" OR "neutrophil lymphocyte ratio" OR "systemic inflammatory response index"):ti,ab,kw (Word variations have been searched)  6. MeSH descriptor: [Disseminated Intravascular Coagulation] explode all trees  7. MeSH descriptor: [Blood Coagulation] explode all trees  8. (coagulopathy OR "sepsis induced coagulopath*" OR "SIC" OR "disseminated intravascular coagulation" OR "DIC" OR "sepsis associated coagulopath*" OR "SAC" OR "coagulation marker*" OR fibrinogen OR prothrombin OR "antithrombin III" OR beta-Thromboglobulin OR "beta Thromboglobulin" OR "von Willebrand Factor*" OR Thrombin OR "Blood Coagulation Factor*" OR "Factor V" OR calcium OR "factor IV" OR "Factor VII" OR "Factor VIII" OR "Factor IX" OR "Factor X" OR "Factor XI" OR "Factor XII" OR "Factor XIII" OR fibrin OR "antifibrinolytic agent*" OR "platelet count" OR "Mean Platelet Volume" OR P-Selectin OR "P Selectin" OR thromboplastin OR "protein C" OR "protein C inhibitor*" OR "protein S" OR thrombomodulin OR "plasminogen inactivator*" OR "plasminogen activator*" OR plasminogen OR carboxypeptidases OR "thrombin-activatable fibrinolysis inhibitor" OR TAFI OR "fibrinopeptide A" OR "fibrinopeptide B" OR hemostat* OR haemostat* OR thrombocyt* OR platelet* OR D-dimer* OR "tissue factor*" OR thrombophilia OR thrombocytopenia OR PT OR "prothrombin time" OR aPTT OR "activated partial thromboplastin time" OR INR OR "international normalised ratio" OR "APTT Ratio" OR "vitamin K" OR "fibrin degradation products" OR FDP OR "fibrin split products" OR FSP OR "Plasminogen activator inhibitor-1" OR PAI-1 OR "Alpha-2 plasmin inhibitor" OR "antiplasmin" OR thromboelasto*):ti,ab,kw (Word variations have been searched)  9. 6 OR 7 OR 8  10. (coagul* AND marker*):ti,ab,kw (Word variations have been searched)  11. 9 OR 10  12. 4 OR 5  13. 11 OR 12  14. MeSH descriptor: [Atrial Fibrillation] explode all trees  15. ("atrial fibrillation" OR "AF" OR "new onset atrial fibrillation" OR new-onset-atrial-fibrillation OR "NOAF" OR "atrial flutter*" OR "supraventricular tachycardia" OR supraventricular-tachycardia OR "supraventricular arrhythmia" OR supraventricular-arrhythmia OR "SVT" OR "atrial tachycardia"):ti,ab,kw  16. 14 OR 15  17. 3 AND 13 AND 16 |
| Scopus | 1. TITLE-ABS-KEY ( sepsis OR septic OR septicemia OR toxemia OR toxaemia OR bacteraemia OR bacteremia )  2. TITLE-ABS-KEY ( inflammation OR inflamm* OR crp OR "C-reactive protein" OR esr OR "Erythrocyte sedimentation rate" OR "plasma viscosity" OR "procalcitonin" OR pct OR il-6 OR il-1 OR "Tumour Necrosis Factor alpha" OR "Serum amyloid A" OR saa OR "heat shock proteins" OR hsps OR tnf OR "Tumour Necrosis Factor" OR il-10 OR "acute phase proteins" OR ferritin OR "soluble IL-2 receptor" OR "white cell count" OR monocyte OR basophil OR lymphocyte OR eosinophil OR neutrophil OR "neutrophil-lymphocyte ratio" OR "neutrophil lymphocyte ratio" OR "systemic inflammatory response index" )  3. TITLE-ABS-KEY ( coagulopathy OR "sepsis induced coagulopath*" OR "SIC" OR "disseminated intravascular coagulation" OR "DIC" OR "sepsis associated coagulopath*" OR "SAC" OR "coagulation marker*" OR fibrinogen OR prothrombin OR "antithrombin III" OR beta-thromboglobulin OR "beta Thromboglobulin" OR "von Willebrand Factor*" OR thrombin OR "Blood Coagulation Factor*" OR "Factor V" OR calcium OR "factor IV" OR "Factor VII" OR "Factor VIII" OR "Factor IX" OR "Factor X" OR "Factor XI" OR "Factor XII" OR "Factor XIII" OR fibrin OR "antifibrinolytic agent*" OR "platelet count" OR "Mean Platelet Volume" OR p-selectin OR "P Selectin" OR thromboplastin OR "protein C" OR "protein C inhibitor*" OR "protein S" OR thrombomodulin OR "plasminogen inactivator*" OR "plasminogen activator*" OR plasminogen OR carboxypeptidases OR "thrombin-activatable fibrinolysis inhibitor" OR tafi OR "fibrinopeptide A" OR "fibrinopeptide B" OR hemostat* OR haemostat* OR thrombocyt* OR platelet* OR d-dimer* OR "tissue factor*" OR thrombophilia OR thrombocytopenia OR pt OR "prothrombin time" OR aptt OR "activated partial thromboplastin time" OR inr OR "international normalised ratio" OR "APTT Ratio" OR "vitamin K" OR "fibrin degradation products" OR fdp OR "fibrin split products" OR fsp OR "Plasminogen activator inhibitor-1" OR pai-1 OR "Alpha-2 plasmin inhibitor" OR "antiplasmin" OR thromboelasto* )  4. TITLE-ABS-KEY ( coagul* AND marker* )  5. 3 OR 4  6. TITLE-ABS-KEY ( "atrial fibrillation" OR "AF" OR "new onset atrial fibrillation" OR new-onset-atrial-fibrillation OR "NOAF" OR "atrial flutter*" OR "supraventricular tachycardia" OR supraventricular-tachycardia OR "supraventricular arrhythmia" OR supraventricular-arrhythmia OR "SVT" OR "atrial tachycardia" )  7. 2 OR 5  8. 1 AND 6 AND 7 |
