# Supplementary material for: A systematic review on the influence of coagulopathy and immune activation on New Onset Atrial Fibrillation in patients with sepsis
Source: PLoS One. 2025 Jan 29;20(1):e0318365. doi: 10.1371/journal.pone.0318365 (PMC11778662; doi:10.1371/journal.pone.0318365)
Supplement: S2 Table — (DOCX) [file pone.0318365.s002.docx]

S2 Table – Patient Characteristics

| Study (author and year of publication) | Age, mean years ± SD ‡ or median (IQR) † | Sex (M/F) | Comorbidities | | Clinical characteristics | | Illness Severity Score | |
| --- | --- | --- | --- | --- | --- | --- | --- | --- |
|  |  |  | Comorbidity | n (%) * | Vital signs | mean ± SD ‡ or median (IQR) † | Scoring System | mean ± SD ‡ or median (IQR) † |
| **Prospective Observational Studies** | | | | | | |  |  |
| Zakynthinos, G. E. et al. (2022) | Controls: 68.2 ± 3.1 ‡ NOAF: 69.7 ± 3.1 ‡ | 60/19 | **Mean±SD:** Charlson comorbidity index  HTN | **Mean±SD:** Control: 3.2 ± 0.4 ‡ NOAF: 3.8 ± 0.88 ‡  Control: 46 /60 (76.6%) * NOAF: 10/19 (52.6%) * | n/r | n/r | APACHE II  SOFA | Control: 16.9 ±1.8 ‡ NOAF: 14.4 ±2.8 ‡  Control: 8 ± 1.3 ‡ NOAF: 7.4 ± 1 ‡ |
| Hayase, N. et al. (2016) | Non-sepsis: 57.2 ± 17.8 ‡ Sepsis:  63.5 ± 15.1 ‡ | 161/106 | ICU admission: Medical critical state     Surgical critical state  Multiple trauma | Nonsepsis:77/172(44.8%) * Sepsis: 74/95 (77.9%) *  Non-sepsis: 77/ 172 (44.8%) * Sepsis: 19/95 (20.0%) *  Nonsepsis:18/172 (10.4%) * Sepsis: 2/95 (2.1%) * | Heart rate (bpm)  New-onset AF/SR **n*** | Non-sepsis: 86.6 ± 1.6 ‡ Sepsis: 101.9 ± 2.1 ‡   Non-sepsis: 5/167(3.00%)* Sepsis:  6/89(6.74%)* | SOFA | Non-sepsis: 5.21 ±3.59 ‡  Sepsis: 7.82 ± 4.27 ‡ |
| Makrygiannis, S. S. et al. (2014) | 52.5 ± 19.6 ‡ | 90/43 | Cause of admission (%): Medical Trauma Nonthoracic surgery History of heart disease: History of lung disease: Smoking  HTN DM Dyslipidaemia  Sleep apnoea IHD  Congenital/  structural HD Respiratory disease Neurological disorders  Rheumatologic disease  Thyroid hormone disorders | 71/133 (53.3%) * 49/133 (36.7%) * 13/133 (10.0%) *  28/133 (21.1%) *  24/133 (18.1%) *  68/133 (51.1%)* 51/133 (38.3%)* 24/133 (18.0%)* 14/133 (10.5%)* 7/133 (5.26%)* 18/133 (13.5%)* 10/133 (7.51%)*  24/133 (18.0%)* 12/133(9.02%)*  3/133 (2.26%)*  5/133 (3.76%)* | n/r | n/r | APACHE II | 16.0 ± 6.6 ‡ |
| Meierhenrich, R. et al. (2010) | Non-septic shock: 67 (46-84) †  Septic shock: 66 (41-85) † | 38/11 | HTN IHD HF Congenital/structural HD Respiratory disease | 33/49 (67.3%)* 8/49 (16.3%))* 1/49 (2.04%)* 1/49 (2.04%)*  8/49 (16.3%)* | n/r | n/r | SOFA  SAPSII | Non-septic shock: 8.5 (4-14) † Septic shock: 12 (7-17) †  Non-septic shock: 34 (7-60) † Septic shock: 31 (15-63) † |
| **Retrospective Observational Studies** | | | | | | | | |
| Li, Z. et al. (2022) | 59.47±16.42 ‡ | 1592/  900 | HTN IHD HF DM Respiratory disease Hyperlipidaemia Stroke Liver failure Renal failure Malignancy | 449/2492 (18.0%) * 234/2492 (9.4%) * 559/2492 (22.4%) *  337/2492 (13.5%) * 186/2492 (7.5%) * 556/2492 (22.3%) * 183/2492 (7.3%) *  199/2492 (8.0%) * 273/2492 (11.0%) * 70/2492 (2.8%) * | HR (bpm)  MAP (mmHg)  BMI (kg/m2) | 105.13 ± 10.25 ‡  96.40 ± 7.01 ‡  22.06 ± 1.89 ‡ | SOFA  APACHEII  SAPSII | 5.00 (3.00–7.00) † 15.00 (10.00–18.00) † 42.00 (36.00–46.00) † |
| Zhai, G. et al. (2021) | 66.1 ± 15.4 ‡ | 2993/2519 | HF IHD Arrhythmias Cardiac arrest Congenital/structural HD Shock HTN DM High Cholesterol Respiratory disease Renal failure Malignancy Stroke Sepsis | 1239/5512 (22.5%) * 3655/5512 (66.4%) * 1841/5512 (33.4%) * 399/5512 (7.2%) * 526/5512 (9.6%) *  1691/5512 (30.7%) * 1665/5512 (30.2%) * 1114/5512 (20.2%) * 395/5512 (7.2%) * 1652/5512 (30.0%) *  1870 /5512 (33.9%) * 267/5512 (4.8%) * 227/5512 (4.1%) * 982/5512 (17.8%) * | Systolic BP (mmHg)  Diastolic BP (mmHg)  Mean BP (mmHg)  Heart rate (beats/min) Respiration rate (breaths/min) Oxygen saturation (%) Body mass index (kg/m2) | 122.6 ± 19.3 ‡  66.1 ± 11.3 ‡  82.2 ± 12.9 ‡  89.2 ± 22.5 ‡  21.1 ± 6.6 ‡  97(95-100) †  29.0 ± 7.5 ‡ | APS  APACHE IV | 41(28-58) † 55(40-72) † |
| Ruiz, L. et al. (2021) | No event: 60.7±17.6 ‡ NOAF: 70.1±12.5 ‡ | 658/434 | Malignancy Liver failure Renal failure Respiratory disease DM Cerebrovascular disease HF IHD HTN Hyperlipidaemia Influenza vaccine Pneumococcal vaccination Nursing home resident  Smoker Heavy drinker | 44/1092(4.02%)* 46/1092(4.21%)* 45/1092(4.12%)* 206/1092(18.9%)* 171/1092(15.7%)* 53/1092(4.85%)*  51/1092(4.67%)* 8/1092(0.73%)* 387/1092(35.4%)* 286/1092(26.2%)* 267/1092(24.5%)* 116/1092(10.6%)*  27/1092(2.47%)*  341/1092(31.2%)* 140/1092(12.8%)* | Mean No of days with symptoms prior to admission (SD)  **All expressed as n: ***  Temp < 35 or >40 °C Altered mental status SBP< 90 mm Hg  DBP ≤ 60 mm Hg Respiratory rate ≥ 30/min | No event: 3.8±2.8 ‡ NOAF:  3.8±2.6 ‡ Total:  6/1092(0.55%)*  103/1092(9.43%)**  106/1092(9.70%)**  383/1092(35.0%)**  229/1092(21.0%)** | PSI >3 | 492/1092  (45.0%)** |
| Long, Y. et al. (2021) | 61 (49–70) † | 4242/3286 | HTN DM HF PVD Renal failure Liver failure Respiratory disease Stroke  DIC | 3,463/7528 (45.98%) * 1,977/7528 (26.26%) * 1,534/7528 (20.39%) * 411/7528 (5.46%) * 1,127/7528 (14.98%) * 1,506/7528 (20.02%) * 1,569/7528 (20.86%) * 89/7528 (1.18%) * 179/7528 (2.38%) * | HR (bpm) RR (bpm)  T (◦C)  MAP (mmHg) BMI (kg/m2) | 90 (78–102) † 19 (17–22) † 36.9 (36.5-37.4) † 77 (70–85) †  27.7 (23.9–33.0) † | ECI  SOFA APSIII  SAPSII  OASIS  GCS | 11 (5-17) † 5 (3-7) † 47(35–62) † 36 (28-46) † 33(27–39) † 15(13–15) † |
| Kanthasamy, V. et al. (2021) | 59(53-65) † | 90/19 | DM HTN IHD High cholesterol Obesity Sleep apnoea HF Renal failure Malignancy | 46/109 (42%) * 61/109 (56%) * 17/109 (16%) * 40/109 (37%) * 25/109 (23%) * 6/109 (6%) * 4/109 (4%) * 92/109 (85%) * 1/109 (1%) * | ECG findings: LV systolic function **n*:** Good ≥55% Mild EF 45-54% Moderate 36-44% Severe ≤35% | 60(55-65) †  83/109 (92%) * 2/109 (2%) *  5/109 (5%) *  1/109 (1%) * | n/r | n/r |
| Bontekoe, J. et al. (2020) | mean: 60 | 48/49 | CKD5-HD AF Sepsis | 97/97 (100%) * 23/97 (23.7%)* 35/97 (36.1%)* | n/r | n/r | n/r | n/r |
| Sun, H. et al.  (2019) | **Age, separated by NLR:** **NLR < 4.80:** 66.8 ± 16.4 ‡  **NLR 4.80–10.08:** 68.5 ± 15.9 ‡  **NLR ≥10.09:** 70.0 ± 14.7 ‡ | 2075/1488 | Sepsis Endocarditis HF AF Renal failure Liver failure IHD Stroke Malignancy Respiratory disease ARDS Pneumonia | 254/3563(7.13%)* 6/3563 (0.17%)* 1059/3563 (29.7%)* 1190/3563 (33.4%)* 3103/3563 (87.1%)* 114/3563 (3.20%)* 1847/3563 (51.8%)* 218/3563 (6.12%)* 379/3563 (10.6%)* 898/3563 (25.2%)* 58/3563 (1.63%)* 861/3563 (24.2%)* | **Separated by NLR:**  HR (beats/min)  Systolic BP (mm Hg)  Diastolic BP (mm Hg)  Mean BP (mm Hg)  RR (beats/min)  T (°C)  SPO 2 ( %)  HR (beats/min)  Systolic BP (mm Hg)  Diastolic BP (mm Hg)  Mean BP (mm Hg)  RR (beats/min)  T (°C)  SPO 2 (%)  HR (beats/min)  Systolic BP (mm Hg)  Diastolic BP (mm Hg)  Mean BP (mm Hg)  RR (beats/min)  T (°C)  SPO 2 ( %) | **NLR < 4.80:**  78.1 ± 16.6 ‡  117.9 ± 17.8 ‡  62.0 ± 11.6 ‡  78.8 ± 11.7 ‡  18.5 ± 3.5 ‡  36.7 ± 0.6 ‡  97.1 ± 2.0 ‡  **NLR 4.80–10.08:**  82.0 ± 16.4 ‡  116.0 ± 17.1 ‡  60.4 ± 11.1 ‡  77.2 ± 11.2 ‡  19.3 ± 3.9 ‡  36.8 ± 0.7 ‡  96.8 ± 2.3 ‡  **NLR ≥10.09**  85.4 ± 17.2 ‡  113.1 ± 16.2 ‡  58.8 ± 10.6 ‡  75.3 ± 10.5 ‡  20.1 ± 4.2 ‡  36.8 ± 0.8 ‡  96.9 ± 2.6 ‡ | APS III | **Separated by NLR: NLR < 4.80:** 39.5 ± 18.2 ‡  **NLR 4.80–10.08:** 43.7 ± 18.1 ‡ **NLR ≥10.09:** 51.9 ± 22.3 ‡ |
| Kindem, Ingvild A. et al. (2008) | Median: 75 NOAF group: 77 mean age **Expressed as n (%) *:** 20-64 yrs: 226/672 (34%) * 65-84 yrs: 331/672 (49%) * >84 yrs:  115/672 (17%) * | 288/384 | S. pneumoniae infection History of paroxysmal AF IHD HF HTN Malignancy Renal failure DM Respiratory disease Heavy drinker | 226/672 (33.6%)*  37/672 (5.51%)*  145/672 (21.6%)* 91/672 (13.5%)* 151/672 (22.5%)* 6/672 (0.89%)* 21/672 (3.13%)* 21/672 (3.13%)* 71/672 (10.6%)* 46/672 (6.85%)* | **Expressed as n*:** Fever  SIRS | 431/672 (64.1%)* 624/672 (92.9%)* | n/r | n/r |
| ‡Mean± Standard Deviation (SD). †Median (Interquartile range). *n (%). ⁱ Mean ± Standard error of the mean (SEM). Comorbidities grouped: ischemic heart disease (IHD) - includes coronary artery disease, acute coronary syndromes, STEMI, NSTEMI, congenital/structural heart disease (HD) - includes valvular disease, congenital HD and cardiomyopathies, respiratory disease - includes COPD, respiratory failure, any other pulmonary conditions, Renal failure - AKI/CKD, Liver failure - acute/chronic. PVD – peripheral vascular disease. DM – diabetes mellitus. HTN – hypertension. ARDS – acute respiratory distress syndrome. Other comorbidities remaining are ungrouped e.g., stroke. NLR – neutrophil lymphocyte ratio. SIRS- systemic inflammatory response syndrome. MAP- mean arterial pressure. APACHE- Acute Physiology and Chronic Health Evaluation. APS – Acute physiology score (SAPS – simplified APS). SOFA - Sequential Organ Failure Assessment. ECI- Elixhauser comorbidity index. PSI – Pneumonia severity index. OASIS – Overall anxiety severity and impairment scale. | | | | | | | | |
