# Supplementary material for: A systematic review on the influence of coagulopathy and immune activation on New Onset Atrial Fibrillation in patients with sepsis
Source: PLoS One. 2025 Jan 29;20(1):e0318365. doi: 10.1371/journal.pone.0318365 (PMC11778662; doi:10.1371/journal.pone.0318365)
Supplement: S3 Table — (DOCX) [file pone.0318365.s003.docx]

S3 Table – Additional Patient Characteristics: Ethnicity, Imaging, Medications and Laboratory Parameters

| Study (author and year of publication) | Ethnicity, n(%)* | Laboratory Parameters ^c^ | | Imaging Results | | Medications | |
| --- | --- | --- | --- | --- | --- | --- | --- |
|  |  | Parameter | mean ± SD ‡ or median (IQR)† | Finding | n(%)* | Usage | n (%)* |
| **Prospective Observational Studies** | | | | | | | |
| Zakynthinos, G. E. et al. (2022) | n/r | PαΟ2/FιΟ2 (mmHg)  CRP  D-Dimers (ng/ml) (< 300)   Ferritin, (ng/ml), (< 330)  WBC, 103/L (< 10 ×103/L)  CRP (mg/dL), (< 0.5)   Troponin, ng/ml (< 0.04) | Control:111.2 ± 45.7 ‡  NOAF:124.6 ± 42.2 ‡ Control:36.7 ± 2.5 ‡ NOAF:37.1 ± 8.9 ‡ Control:819 ± 398.5 ‡ NOAF:895 ± 353.6 ‡ Control:1205.7 ±  952.8 ‡ NOAF:1380 ± 801.5 ‡ Control:9273.2 ± 6498 ‡ NOAF:9543.8 ± 2108‡ Control:8.4 ± 0.4 ‡ NOAF:10.9 ± 4.2 ‡ Control:0.15 ± 0.34 ‡ NOAF:0.16 ± 0.31 ‡ | PE | 1/60(1.7%)* control | Corticosteroids    **Dose mean±SD:** Noradrenaline (μg/kg/min) | Control:  54/60 (84%)* ^a^ NOAF: 14/19 (74%)* ^a^  Control: 0.39 ± 0.18 ‡ ^a^ NOAF: 0.24 ± 0.13 ‡ ^a^ |
| Hayase, N. et al. (2016) | n/r | Serum creatinine (mg/dL)  PaO2/FiO2   NT-proBNP (pg/mL)  NGAL (ng/mL) | Non: 0.72 (0.55–0.99)†  Sepsis:1.08 (0.64–1.72)† Non:385 (274–483) † Sepsis:230 (139–357)† Non:120 (41–424)† Sepsis:1,070 (242–3,010)† Non: 65.5 (44.2–122)† Sepsis: 327 (112–542)† | n/r | n/r | Cont. Sedation  Cont. Analgesia Beta Blocker  Digoxin  Calcium channel blocker | 172/172 (100%)* ^b^ 127/172 (73.8%)* ^b^ 11/172(6.40%)* ^b^ 2/172(1.16%)* ^b^ 5/172(2.91%)* ^b^ |
| Makrygiannis, S. S. et al. (2014) | n/r | n/r | n/r | PE  Left ventricle EF <0.35  New regional systolic dysfunction Diastolic dysfunction  Left ventricle hypertrophy Left atrium dilatation  Pericardial effusion  Valvular disorders  Pulmonary HTN *****(Echocardiography data total n=119)***** | 2/20 (10.0%)* NOAF group 11/119 (9.24%)*  3/119 (2.52%)*  36/119 (30.3%)*  13/119 (10.9%)*  18/119 (15.1%)*  8/119 (6.72%)*  15/119 (12.6%)*  14/119 (11.8%)* ******* | n/r | n/r |
| Meierhenrich, R. et al. (2010) | n/r | n/r | n/r | n/r | n/r | Beta blocker  Digitalis  Calcium antagonist ACE inhibitor | 16/49 (32.7%)* ^a^  1/49 (2.04%)* ^a^ 12/49(24.5%)* ^a^  12/49(24.5%)* ^a^ |
| **Retrospective Observational Studies** | | | | | | | |
| Li, Z. et al. (2022) | n/r | WBC count (× 10^9/L) Haemoglobin (g/L)  PLT count (×10^9/L) Platelet distribution width (%) Serum creatinine (μmol/L) BUN (mmol/L) ALT (U/L) Bilirubin (μmol/L) Albumin (g/L) Cardiac troponin I (ng/mL)  BNP (pg/mL) APTT (s) PT (s) INR Fibrinogen (g/L) D-dimer (mg/L) Lactic acid (mmol/L) Procalcitonin (μg/L) CRP (mg/L) | 13.40 (12.00–14.60)† 114.00 (111.00–117.00)† 156.0 (98.00–164.00)† 16.10 (15.40–16.80)†  80.44 (73.23–86.67)† 7.20 (5.70–8.40)† 35.00 (24.00–47.00)† 25.03 (21.80–28.49)† 39.84 (34.73–44.82)† 0.05 (0.04–0.06)† 94.42 (80.90–108.93)† 35.20 (31.62–38.70)† 15.20 (13.70–17.40)†  1.28 (1.10–1.72)† 4.06 (3.69–4.44)† 2.92 (1.62–6.39)† 4.40 (3.69–5.11)† 3.03 (2.70–3.40)† 46.00 (17.92–89.36)† | n/r | n/r | Corticosteroid | 583/2492 (23.4)* ^b^ |
| Zhai, G. et al. (2021) | Caucasian 3914/5512 (70.0%)* African American 896/5512 (16.3%)*  Other 702/5512 (12.7%)* | WBC (10^9/L)  Lymphocyte (%)  Monocyte (%)  Neutrophil (%) RBC (10^9/L)  Platelet (10^9/L)  Haemoglobin (g/dL) Haematocrit (%) Glucose (mg/dL) Creatinine (mg/dL)  BUN (mg/dL) Sodium (mmol/L) Potassium (mmol/L)  MLR | 11.6 ± 5.6 ‡  15.4 ± 10.9 ‡ 7.6 ± 3.6 ‡ 74.7 ± 12.4 ‡ 4.1 ± 0.8 ‡ 230 ± 96 ‡ 12.0 ± 2.5 ‡ 36.4 ± 7.1 ‡ 165.0 ± 102.9 ‡ 1.79 ± 1.71 ‡ 29.0 ± 19.1 ‡ 136.8 ± 7.4 ‡ 4.2 ± 0.8 ‡ 0.57(0.34- 0.96)† | n/r | n/r | Antiplatelet  Oral anticoagulants  Beta Blocker  ACEI/ARB  Statins | 2630/5512 (47.7%)* ^a^ 683/5512 (12.4%)* ^a^  2407/5512 (43.7%)* ^a^ 1457/5512 (26.4%)* ^a^ 1684/5512 (30.6%)* ^a^ |
| Ruiz, L. et al. (2021) | n/r | **All expressed as n:*** BUN ≥ 30 mg/dL PaO2 < 60 mm Hg  Glucose>250 mg/dL  Haematocrit < 30%  Blood pH < 7.35  Leukocyte count < 4000 (x10^9 /L)  Inflammation(CRP&WBC): Mild  Moderate  Severe +++ urinary antigen test  +++ blood culture | 384/1092(35.2%)* 460/1092(42.1%)** 83/1092(7.60%)** 35/1092(3.21)** 69/1092(6.32%)** 50/1092(4.58%)**   226/1092(20.7%)** 287/1092(26.3%)** 579/1092(53.0%)** 952/1092(87.2%)* 460/1092(42.1%)* | Multilobar Pneumonia  Pleural Effusion | 308/1092(31.3%)*  107/1092(10.9%)* | Prior antibiotic treatment | 104/1092  (9.52%)* ^a^ |
| Long, Y. et al. (2021) | Asian 225/7528 (2.99%)*  Black 773/7528 (10.27%)* Hispanic 316/7528 (4.20%)* White 5,333/7528 (70.84%)* Other 881/7528 (11.70%)* | PLT(10^9/L) INR APTT(s) | 179 (109–256) †  1.3 (1.1–1.7) †  31.5 (27–40.5)† | n/r | n/r | n/r | n/r |
| Kanthasamy, V. et al. (2021) | n/r | n/r | n/r | Venous arterial thrombo-embolism  PE diagnosis | 24/109 (22)*  18/24 (76%) | n/r | n/r |
| Bontekoe, J. et al. (2020) | n/r | **Separated by control, CKD +AF and CKD-AF:** PCT (pg/mL)  Ang-1 (ng/mL)  Ang-2 (ng/mL)  CRP (mg/mL)  CD40-L (ng/mL)  D-dimer (mg/mL)  TNF- a (pg/mL)  vWF (%)   PCT (pg/mL)  Ang-1 (ng/mL)  Ang-2 (ng/mL)  CRP (mg/mL)  CD40-L (ng/mL)  D-dimer (mg/mL)  TNF- a (pg/mL)  vWF (%)   PCT (pg/mL)  Ang-1 (ng/mL)  Ang-2 (ng/mL)  CRP (mg/mL)  CD40-L (ng/mL)  D-dimer (mg/mL)  TNF- a (pg/mL)  vWF (%) | **Controls:** 17.64 ± 3.20 ⁱ 0.11 ± 0.08 ⁱ 1.87 ± 0.15 ⁱ 1.24 ± 0.28 ⁱ 0.08 ± 0.01 ⁱ 0.08 ± 0.01 ⁱ 0.14 ± 0.07 ⁱ 93.2 ± 2.68 ⁱ **CKD5-HD +AF:** 113.8 ± 13.9 ⁱ 597.9 ± 84.1 ⁱ 9.61 ± 0.70 ⁱ 8.12 ± 1.13 ⁱ 304.1 ± 36.3 ⁱ 1069.8 ± 145.7 ⁱ 8.67 ± 2.40 ⁱ 132.3 ± 2.92 ⁱ **CKD5-HD -AF:** 143.6 ± 25.3 ⁱ 690.9 ± 169.7 ⁱ 13.05 ± 1.56 ⁱ 12.11 ± 2.41 ⁱ 350.3 ± 102.3 ⁱ 1249.7 ± 402.9 ⁱ 3.92 ± 0.99 ⁱ 134.8 ± 5.28 ⁱ | n/r | n/r | n/r | n/r |
| Sun, H. et al.  (2019) | White 2553/3563  (71.7%)* Black 269/3563  (7.55%)* Other 741/3563  (20.8%)* | **Separated by NLR:** Neutrophils(n (%)) Lymphocytes (n (%)) Anion gap(mmol/l) Bicarbonate(mmol/l) Chloride(mmol/l) Glucose (mg/dl) Potassium(mmol/l) Sodium (mmol/l) Haematocrit(%) Haemoglobin (g/dl) Platelet count (10^9/l) WBC count (10^9/l) Creatinine(mg/dl) BUN (mg/dl)   Neutrophils(n (%)) Lymphocytes (n (%)) Anion gap(mmol/l) Bicarbonate(mmol/l) Chloride(mmol/l) Glucose (mg/dl) Potassium(mmol/l) Sodium (mmol/l) Haematocrit(%) Haemoglobin (g/dl) Platelet count (10^9/l) WBC count (10^9/l) Creatinine(mg/dl) BUN (mg/dl)   Neutrophils(n (%)) Lymphocytes (n (%)) Anion gap(mmol/l) Bicarbonate(mmol/l) Chloride(mmol/l) Glucose (mg/dl) Potassium(mmol/l) Sodium (mmol/l) Haematocrit(%) Haemoglobin (g/dl) Platelet count (10^9/l) WBC count (10^9/l) Creatinine(mg/dl) BUN (mg/dl) | **NLR < 4.80:** 64.9 ± 12.4 ‡ 26.3 ± 10.9 ‡ 13.1 ± 3.2 ‡ 23.0 ± 4.3 ‡ 101.8 ± 5.1 ‡ 114.1 ± 38.4 ‡ 3.8 ± 0.5 ‡ 136.9 ± 4.2 ‡ 32.7 ± 6.2 ‡ 11.2 ± 2.2 ‡ 212.8 ± 92.7 ‡ 8.6 ± 6.0 ‡ 1.3 ± 1.4 ‡ 23.7 ± 18.6 ‡  **NLR 4.80–10.08:** 81.3 ± 5.2 ‡ 11.8 ± 2.3 ‡ 13.6 ± 3.2 ‡ 22.5 ± 4.8 ‡ 101.4 ± 5.7 ‡ 120.4 ± 41.5 ‡ 3.8 ± 0.5 ‡ 136.5 ± 4.9 ‡ 32.0 ± 6.2 ‡ 10.9 ± 2.2 ‡ 215.9 ± 101.2 ‡ 10.2 ± 4.1 ‡ 1.5 ± 1.4 ‡ 28.1 ± 19.8 ‡  **NLR ≥10.09:** 88.3 ± 5.8 ‡ 5.4 ± 2.0 ‡ 14.2 ± 3.5 ‡  21.5 ± 5.6 ‡ 101.1 ± 6.5 ‡ 124.8 ± 46.8 ‡ 3.9 ± 0.6 ‡ 136.2 ± 5.2 ‡  31.2 ± 5.6 ‡ 10.6 ± 1.9 ‡ 230.8 ± 113.1 ‡ 13.2 ± 6.5 ‡ 1.6 ± 1.4 ‡  33.6 ± 23.6 ‡ | n/r | n/r | Vasoactive drugs | 1219/3563 (34.2%)* ^a^ |
|  |  |  |  |  |  |  |  |
| Kindem, Ingvild A. et al. (2008) | n/r | n/r | n/r | n/r | n/r | Corticosteroids prior to admission | 34/672 (5.06%)* ^a^ |
| ^a^ Medications data has been extracted from study baseline characteristics & demographics, only medications listed above were reported (see patient outcomes table for data concerning our secondary outcome: vasopressor usage). ^b^ Treatment data for non-vasopressor drugs (see patient outcomes for vasopressor usage). ^c^ All laboratory parameters reported and extracted from baseline characteristics. ‡Mean± Standard Deviation (SD). †Median (Interquartile range). *n (%). ⁱ Mean ± Standard error of the mean (SEM). CRP- C reactive protein. WBC – white blood cell count. NGAL-Neutrophil gelatinase-associated lipocalin. BNP- brain natriuretic peptide. BUN- blood urea nitrogen. PE- pulmonary embolism. | | | | | | | |
