# Supplementary material for: A systematic review on the influence of coagulopathy and immune activation on New Onset Atrial Fibrillation in patients with sepsis
Source: PLoS One. 2025 Jan 29;20(1):e0318365. doi: 10.1371/journal.pone.0318365 (PMC11778662; doi:10.1371/journal.pone.0318365)
Supplement: S4 Table — (DOCX) [file pone.0318365.s004.docx]

S4 Table – Quality of Methodology (risk of bias)

| Author(s) | Year of publication | Selection | | | | Comparability | Outcome | | | Total (maximum of 9 stars) |
| --- | --- | --- | --- | --- | --- | --- | --- | --- | --- | --- |
|  |  | Representa-tiveness of exposed patients | Selection of non-exposed patients | Ascertain-ment of exposure | Demonstra-tion that the outcome of interest was not present initially | Patient cohorts controlled for confounders | Ascertain-ment of outcome | Was follow-up long enough? | Adequacy of follow-up cohorts |  |
|  |  |  |  |  |  |  |  |  |  |  |
| Zhai, G. et al. | 2021 |  |  | * |  | ** | * | * |  | 5  (low) |
| Zakynthinos, G. E. et al. | 2022 |  | * | * | * | * | * | * | * | 7  (moderate) |
| Ruiz, L. et al. | 2021 |  | * | * | * | * | * | * | * | 7  (moderate) |
| Makrygiannis, S. S. et al. | 2014 |  | * | * | * | * | * | * |  | 6  (low) |
| Long, Y. et al. | 2021 | * | * | * |  | ** | * | * | * | 8  (high) |
| Li, Z. et al. | 2022 | * | * | * | * | ** | * | * |  | 8  (high) |
| Kindem, Ingvild A. et al. | 2008 |  | * | * |  | * | * | * | * | 6  (low) |
| Kanthasamy, V. et al. | 2021 |  | * | * | * | * | * | * | * | 7  (moderate) |
| Hayase, N. et al. | 2016 |  | * | * | * | ** | * | * | * | 8  (high) |
| Sun, H. et al. | 2019 |  | * | * |  | ** | * | * | * | 7  (moderate) |
| Meierhenrich, R. et al. | 2010 |  | * | * | * | * | * | * |  | 6  (low) |
| Bontekoe, J. et al. | 2020 |  |  | * |  | * | * |  |  | 3  (low) |
| ≤6 low methodological quality. 7 = moderate methodological quality. ≥8 high methodological quality. | | | | | | | | | | |
