# Supplementary material for: A systematic review on the influence of coagulopathy and immune activation on New Onset Atrial Fibrillation in patients with sepsis
Source: PLoS One. 2025 Jan 29;20(1):e0318365. doi: 10.1371/journal.pone.0318365 (PMC11778662; doi:10.1371/journal.pone.0318365)
Supplement: S5 Table — (DOCX) [file pone.0318365.s005.docx]

S5 Table – Additional Patient Outcomes

| Study (author and year of publication) | AF (general not necessarily NOAF) | Mortality | ICU admission | LOS hospital | LOS  ICU | Acute renal failure | Need for RRT |
| --- | --- | --- | --- | --- | --- | --- | --- |
| **Prospective Observational Studies** | | | | | | | |
| Zakynthinos, G. E. et al. (2022) | n/r | **28th day mortality** 9/19 (47%)* vs 25/60 (41.7%)*  NOAF vs control | All ICU patients | n/r | n/r | n/r | n/r |
| Hayase, N. et al. (2016) | n/r | NOAF on 28-day survival was not significant (yet tachycardia was significant). **HR(CI)**:  4.22 (1.10–27.72) ^a^  p=-0.034 | Entire cohort ICU based | n/r | n/r | n/r | n/r |
|  |  |  |  |  |  |  |  |
| Makrygiannis, S. S. et al. (2014) | n/r | n/r | All ICU patients | n/r | n/r | n/r | n/r |
| Meierhenrich, R. et al. (2010) | n/r | **Mortality rates NOAF+sepsis** =10/23 (43.5%)* **SR+sepsis** = 6/27 (22.2%)*  **NOAF, no sepsis**= 4/26 (15.4%)*  **ICU-mortality** **NOAF, no sepsis**= 4/26 (15%)* p=0.06 **NOAF+sepsis**= 10/23(44%)* p=0.14  **SR+Sepsis**= 6/27 (22%)* **28-day mortality  NOAF, no sepsis**= 4/26 (15%)* p=0.10 **NOAF+sepsis**= 9/23(39%)* p= 0.22  **SR+sepsis**= 6/27 (22%)* **60-day mortality NOAF, no sepsis**= 6/26 (23%)* p=0.08  **NOAF+sepsis=** 11/23 (48%)* p=0.14 **SR+sepsis=**7/27 (26%)*  **Two year follow up not significant p=0.075** | ICU cohort | n/r | **ICU length of stay, days** **(n=survivors)** **NOAF+no sepsis** 10.5 (2-45)† (n = 22) **NOAF+sepsis** *p< 0.001  30 (9-123)† (n = 13) *p=0.017 **SR+sepsis** 17 (4-48)† (n = 21)  *p values compare NOAF, no sepsis to NOAF, sepsis and NOAF, sepsis to SR sepsis. | n/r | n/r |
| **Retrospective Observational Studies** | | | | | | | |
| Li, Z. et al. (2022) | n/r | **In-hospital mortality, n(%)** ALL= 538/2492 (21.6%)*  Non NOAF= 457/2223 (20.6%)* NOAF=81/269 (30.1%)* X^2=12.938  p<0.001 | **Readmission only No. (%)** All= 345/2492 (13.8%)* Non NOAF = 299/2223 (13.5%)*  NOAF= 46/269(17.1%)* X^2= 2.680  p=0.102 | median (IQR),days All = 12.00 (7.00–18.00)† Non NOAF= 11.00 (7.00–18.00)† NOAF= 13.00 (8.00–21.00) † X^2 =2.247  p=0.025 | median (IQR), days  All= 2.00 (2.00–4.00) † Non NOAF= 2.00 (2.00–4.00) † NOAF = 4.00 (2.00–6.00)† X^2= 8.915  p<0.001 | n/r | n/r |
| Zhai, G. et al. (2021) | 1841/5512* had AF  **ORs (mortality and MLR subgroup analysis) grouped by MLR quartiles:** **MLR < 0.34:**  Reference ^a^  **0.34 ≤ MLR < 0.57:** 1.29(0.68–2.41) ^a^ **0.57 ≤ MLR < 0.96:** 1.48(0.82–2.70) ^a^ **MLR ≥ 0.96:** 2.10(1.19–3.71) ^a^ [p=0.505] | In hospital mortality n(%) = 602/5512 (10.9)*  Quartile 1 =109/1404 (7.8%) * Q2 = 107/1327 (8.1%) * Q3 = 162/1408(11.5%) * Q4=224/1373(16.3%)* [p<0.001] **ORs by quartile** **Quartile 1: MLR < 0.34** Reference ^a^ **Q2: 0.34 ≤ MLR < 0.57**  1.17(0.84–1.64) ^a^ p=0.360  **Q3: 0.57 ≤ MLR < 0.96**  1.47(1.07–2.01) ^a^ p=0.016  **Q4: MLR ≥ 0.96** 1.87(1.38–2.56) ^a^ p<0.001 | 5512 CICU patients (total) | median(IQR) (days)  Total= 6.3(3.9, 11.2)†  Q1: 5.3(3.1, 9.3)† Q2:5.7(3.6, 10.0)†  Q3: 7.0(4.2, 11.9)†  Q4: 8.3(4.8, 11.1)† p=<0.001 | Median (IQR) (days) Total= 2.3(1.4, 4.3)†  Q1= 2.1(1.2, 3.7) † Q2 =2.1(1.2, 3.9)†  Q3= 2.5(1.5, 4.6)† Q4= 2.8(1.7, 5.4)† p=<0.001 | 1019/5512 (18.5%)*  AKIs **ORs (mortality and MLR) by quartile: Q1:** Reference^a^ **Q2:** 0.98(0.60–1.59)^a^  **Q3:** 1.08(0.69–1.69)^a^  **Q4:** 1.17(0.76–1.80)^a^ p=<0.001 | n/r |
| Ruiz, L. et al. (2021) | Focused on NOAF, prior AF excluded for study | Overall 48/1092 (4.4%)* died (all cause)  Mortality rate for those who developed NOAF vs no event  20/109 (17.9%)* vs 29/983 (2.9%)* p < 0.001  Mortality rate for Persistent vs paroxysmal NOAF 11/31 (34.8%)* vs 5/78 (6.3%)*, p = 0.002 | Those with paroxysmal AF were most likely to be admitted to ICU but no values given (cannot be accurately determined from graph). | Mean LOS NOAF vs no event 9* vs 6* days, p < 0.001 | n/r | n/r (renal disease stated) | n/r |
| Long, Y. et al. (2021) | AF 1243/7528(16.5%)* Non AF = 6285/7528(83.5%)* | **ICU morality, n (%)** total 785/7528 (10.43%)* No AF 626/6285 (9.96%)* AF 159/1243 (12.79%)*  p=0.003 **In-hospital mortality** total 1,112/7528 (14.77%)* No AF 867/6285 (13.79%)*  AF 245/1243 (19.71%) * p<0.001 **28-day mortality** total 1,236/7528 (16.41) * No AF 968/6285 (15.40) * AF 268/1243 (21.56) * p<0.001 **90-day mortality** total 4,528/7528 (56.56) * No AF 3,522/6285 (56.04)* AF 736/1243 (59.21) * p=0.001 | **OR(CI) for AF by ICU type**  **SICU = 1 ref** ^a^ **TSICU**  0.875; (0.677–1.130) p=0.306 ^a^ 0.951; (0.689–1.311) p=0.758 **(PSM)** ^a^ **MICU**  0.871; (0.726–1.044)  p=0.135 ^a^ 0.814; (0.649–1.019) p=0.073 **(PSM)** ^a^ | n/r | n/r | Renal failure  **OR (CI) of AF** 1.459;(1.203–1.769), p<0.001 ^a^ 1.599; (1.254–2.040) p<0.001 **(PSM) ^a^** | n/r |
| Kanthasamy, V. et al. (2021) | n/r | **In-hospital death:** 38/109(35%)* total 11/16(69%)* NOAF  27/93(29%)* non- NOAF p=0.002  **OR of mortality with NOAF** 5.4; (95% CI 1.7-17); ^a^ p=0.004 | Entire cohort ICU based | Entire cohort ICU based | median (IQR) days **Survivors:** total 35(22-42)† NOAF 42(37-44)† non NOAF 32(21-40)† p=0.03 **Non-survivors:** total 17(12-22)† NOAF 18(12-26)† non NOAF 15(12-21)† p=0.39 | 70/109 (64%)* had an AKI AKI more common with NOAF 15/16 (94 %)* vs 55/93 (59%)*;  p=0.028 | 25/70 (36%) * required RRT |
| Bontekoe, J. et al. (2020) | Comorbid AF in 23/97 (23.7%) * patients | n/r | n/r | n/r | n/r | CKD5-HD cohort | n/r |
| Sun, H. et al.  (2019) | AF= 1190/3563(33.4%)* No AF= 2372/3563(66.6%)* **ORs for mortality by NLR (subgroups)  4.80** AF = 1 ref ^a^ No AF = 1 ref ^a^ **4.80–10.08** AF =1.41 (0.98, 2.04) ^a^ No AF= 1.49 (1.10, 2.01) ^a^   **≥10.0** AF=2.24 (1.59, 3.15) ^a^ No AF=2.75 (2.09, 3.61) ^a^ p=0.5043 | **30 day (all cause) by NLR < 4.80** Ref value=1 ^a^ **4.8–10.08** adjusted= 1.37 (1.08, 1.73) ^a^ p=0.0102 **≥10.09** adjusted= 1.45 (1.16, 1.83) ^a^ p=0.0013 **90 day (all cause)  4.80** ref value =1 ^a^ **4.8–10.08**  adjusted= 1.41 (1.15, 1.72) ^a^ p=0.0010 **≥10.09** adjusted= 1.51 (1.24, 1.84)^a^  p< 0.0001 | Entire cohort CCU | Entire cohort CCU | n/r | AKI=2473/3563 (69.4%) * no AKI=1089/3563 (30.6%)* **ORs for NLR and mortality (subgroup) < 4.80** ref=1 ^a^ **4.80–10.08** AKI= 1.35 (1.04, 1.77)^a^ no AKI= 1.64 (1.02, 2.64) ^a^ **≥10.09** AKI=2.12 (1.66, 2.72)^a^ no AKI= 3.75 (2.42, 5.81)^a^ p=0.0344 | n/r |
|  |  |  |  |  |  |  |  |
| Kindem, Inglvild A. et al. (2008) | n/r | **HR for in hospital mortality AF vs Non AF** 2.25 (95% CI: 1.37–3.68;  p = 0.001)^a^ Adjusted ratio: 1.63 (95% CI: 0.921–2.87; p = 0.093) ^a^ Overall deaths: 46/672 (6.85%)* in 4 days and 78/672 (11.6%)* in 2 weeks | n/r | n/r | n/r | n/r (Only chronic renal failure) | n/r |
| *n(%) ‡ Mean ± standard deviation (SD) † Median (Interquartile range - IQR) a Odds Ratio/Hazard ratio/Regression (confidence interval 95%). PSM – propensity score matching. TSICU – trauma surgical intensive care unit. SICU – surgical ICU. MICU – medical ICU. SR – sinus rhythm. CKD5-HD – chronic kidney disease stage 5 -haemodialysis. RRT – renal replacement therapy. AKI – acute kidney injury. | | | | | | | |
