# Supplementary material for: A systematic review on the influence of coagulopathy and immune activation on New Onset Atrial Fibrillation in patients with sepsis
Source: PLoS One. 2025 Jan 29;20(1):e0318365. doi: 10.1371/journal.pone.0318365 (PMC11778662; doi:10.1371/journal.pone.0318365)
Supplement: S6 Table — (DOCX) [file pone.0318365.s006.docx]

S6 Table – Reporting of vasopressor usage (secondary outcome)

| Study (author and year of publication) | Vasopressor therapy |
| --- | --- |
| **Prospective Observational Studies** | |
| Zakynthinos, G. E. et al. (2022) | **NA usage**  0.08 ± 0.06 μg/kg/min - 3 days before NOAF‡  0.44 ± 0.22 μg/kg/min‡ - day of NOAF occurrence p=0.01  0.52 ± 0.06 μg/kg/min‡, just after NOAF occurrence, p=0.033 |
| Hayase, N. et al. (2016) | **Usage n (%)**  Norepinephrine = 60/267 (22.3%)*  Epinephrine = 1/267 (0.37%)*  Dobutamine = 2/267 (0.75%)*  Dopamine = 11/267 (4.11%)*  **HR for 28-day survival in non-patients with sepsis**  **NA**  2.12(0.27–11.76) a p=0.44  **Dopamine**  0.40(0.022–5.84) ^a^ p=0.51  **28-day mortality comparison**  **Epinephrine**  **Non sepsis non-survivor**  1/11 (9.1%) * p=0.064  **Norepinephrine**  **non sepsis**  non survivor= 4/11 (36.4%) * vs survivor= 8/116 (5.0%) * p=0.004  **Sepsis**  non= 8/14 (57.1%) * vs survivor= 40/81 (49.4%) * p=0.77  **Dopamine**  **non-sepsis**  non survivor = 2/11 (18.2%) * vs survivor= 2/116 (1.2%) *p=0.021  **Sepsis**  Non survivor = 0* vs survivor= 2/81 (2.5%) *p=1.00  **Dobutamine**  **Non-sepsis**  Non survivor= 0* vs survivor= 0*  **Sepsis**  non= 2/14 (14.3%) * vs survivor = 5/81 (6.2%) *p=0.27  **Linear regression for independent determinants of NT-proBNP**  **Univariate**  **Norepinephrine**  0.36 (0.01–0.71) ^a^ p=0.044  **Dopamine**  0.25(-0.44 to 0.94) ^a^ p=0.47  **Dobutamine**  0.94(-0.31 to 2.18) ^a^ p= 0.14  **Multivariate**  **Norepinephrine**  0.18(-0.11 to 0.49) ^a^ p=0.23 |
| Meierhenrich, R. et al. (2010) | **Noradrenaline max. (μg/kg/min)** NOAF, no sepsis= 0.18 (0.00-1.00) † p< 0.01  NOAF+ sepsis= 0.50 (0.15-2.00) † p=0.13 SR+sepsis= 0.30 (0.15-1.40)† **Noradrenaline at AF (μg/kg/min)** NOAF, no sepsis= 0.05 (0.00-0.40)† NOAF+sepsis=0.40 (0.03-1.10) † p< 0.01 **Dobutamine (n%)** NOAF, no sepsis= 2/26 (7.69%)* p< 0.01  NOAF+sepsis= 10/23 (38.4%)* p=0.14 SR+ sepsis= 6/27(22.2%) * |
| **Retrospective Observational Studies** | |
| Li, Z. et al. (2022) | **Dopamine OR (CI) for NOAF** 1.876(1.227- 2.874) ^a^ p=0.004  **Usage n (%)** Epinephrine = 136/2492 (5.5%) * Norepinephrine = 578/2492 (23.2%) * Dopamine = 538/2492 (21.6%) * |
| *n (%) ‡ Mean ± standard deviation (SD) † Median (Interquartile range) ^a^ Odds Ratio/Hazard ratio/Regression (confidence interval 95%). Studies not listed within this table did not report vasopressor usage. SR- sinus rhythm. BNP – brain natriuretic peptide. | |
