# Supplementary material for: A systematic review on the influence of coagulopathy and immune activation on New Onset Atrial Fibrillation in patients with sepsis
Source: PLoS One. 2025 Jan 29;20(1):e0318365. doi: 10.1371/journal.pone.0318365 (PMC11778662; doi:10.1371/journal.pone.0318365)
Supplement: S7 Table — (DOCX) [file pone.0318365.s007.docx]

S7 Table - Inflammatory and Coagulation Biomarkers

| Study (author and year of publication) | CRP | WBC | D-Dimer | Ferritin | PLT | Troponin  I | Troponin  T | Mono | L  Y  M | NE | INR | APTT | NT-Pro  BNP | Fibrinogen | PCT |
| --- | --- | --- | --- | --- | --- | --- | --- | --- | --- | --- | --- | --- | --- | --- | --- |
| **Prospective Observational Studies** | | | | | | | | | | | | | | | |
| Zakynthinos, G. E. et al.  (2022) | ✓ | ✓ | n/r | ✓ | n/r | ✓ | n/r | n/r | n/r | n/r | n/r | n/r | n/r | n/r | n/r |
| Hayase, N. et al.  (2016) | n/r | n/r | n/r | n/r | n/r | n/r | n/r | n/r | n/r | n/r | n/r | n/r | ✓ | n/r | n/r |
| Makrygiannis, S. S. et al. (2014) | ✓ | n/r | n/r | n/r | n/r | n/r | n/r | n/r | n/r | n/r | n/r | n/r | n/r | n/r | n/r |
| Meierhenrich, R. et al.  (2010) | ✓ | n/r | n/r | n/r | n/r | n/r | n/r | n/r | n/r | n/r | n/r | n/r | n/r | n/r | n/r |
| Retrospective Observational Studies | | | | | | | | | | | | | | | |
| Li, Z. et al.  (2022) | ✓ | n/r | n/r | n/r | n/r | n/r | n/r | n/r | n/r | n/r | ✓ | n/r | n/r | ✓ | n/r |
| Zhai, G. et al.  (2021) | n/r | ✓ | n/r | n/r | ✓ | n/r | n/r | n/r | n/r | ✓ | n/r | n/r | n/r | n/r | n/r |
| Ruiz, L. et al.  (2021) | ✓ | ✓ | n/r | n/r | n/r | n/r | n/r | n/r | n/r | n/r | n/r | n/r | n/r | n/r | n/r |
| Long, Y. et al.  (2021) | n/r | n/r | n/r | n/r | ✓ | n/r | n/r | n/r | n/r | n/r | ✓ | ✓ | n/r | n/r | n/r |
| Kanthasamy, V. et al. (2021) | ✓ | ✓ | ✓ | ✓ | n/r | n/r | ✓ | n/r | n/r | n/r | n/r | n/r | n/r | n/r | n/r |
| Bontekoe, J. et al. (2020) | ✓ | n/r | ✓ | n/r | n/r | n/r | n/r | n/r | n/r | n/r | n/r | n/r | n/r | n/r | ✓ |
| Sun, H. et al.  (2019) | n/r | n/r | n/r | n/r | n/r | n/r | n/r | n/r | n/r | n/r | n/r | n/r | n/r | n/r | n/r |
| Kindem, Inglvild A. et al.  (2008) | ✓ | n/r | n/r | n/r | n/r | n/r | n/r | n/r | n/r | n/r | n/r | n/r | n/r | n/r | n/r |
| CRP = C-reactive protein, WBC = white blood cell count, PLT = platelet count, Mono = monocyte count, LYM = lymphocyte count, NE = neutrophil count, INR = international normalised ratio, APTT = activated partial thromboplastin time, NT-ProBNP = N-terminus – Pro- Brain Natriuretic peptide, PCT = procalcitonin. n/r = not reported. ✓ = reported. | | | | | | | | | | | | | | | |
